# The decline of vitamin D in adolescent athletes: insights from a one-year follow-up and its association with electrocardiographic changes

**DOI:** 10.3389/fcvm.2025.1731919

**Published:** 2025-12-08

**Authors:** Mahmoud Mohammed Ramadan, Abdallah A. Jaber, Ahmad J. Aladwani, Abdulrahman E. Alayyaf, Arkan Sam Sayed-Noor, Heba Bassiony Ghanem, Mohamed El-Sherbiny, Hasnaa Ali Ebrahim, Mona Mohamed Ahmed, Dalia Mahmoud Abdelmonem Elsherbini

**Affiliations:** 1 Department of Clinical Sciences, College of Medicine, University of Sharjah, Sharjah, United Arab Emirates; 2 Department of Cardiology, Faculty of Medicine, Mansoura University, Mansoura, Egypt; 3 Department of Paediatrics, Al-Kuwait Hospital, Sharjah, United Arab Emirates; 4 Department of Clinical Laboratory Sciences, College of Applied Medical Sciences, Jouf University, Sakaka, Saudi Arabia; 5 Department of Medical Biochemistry, Faculty of Medicine, Tanta University, Tanta, Egypt; 6 Department of Basic Medical Sciences, College of Medicine, AlMaarefa University, Riyadh, Saudi Arabia; 7 Research Center, Deanship of Scientific Research and Post-Graduate Studies, AlMaarefa University, Riyadh, Saudi Arabia; 8 Department of Basic Medical Sciences, College of Medicine, Princess Nourah bint Abdulrahman University, Riyadh, Saudi Arabia; 9 Department of Physiology, Faculty of Medicine, Cairo University, Cairo, Egypt; 10 Department of Biophysiology, Ibn Sina National College for Medical Studies, Jeddah, Saudi Arabia; 11 Department of Anatomy, Faculty of Medicine, Mansoura University, Mansoura, Egypt

**Keywords:** adolescent, athletes, vitamin D, electrocardiogram, cardiovascular assessment

## Abstract

**Introduction:**

Annual health evaluations are essential for athletes to ensure fitness for competition and enable the early detection of emerging health issues.

**Aim:**

This research aimed to evaluate the longitudinal variations in vitamin D concentrations and associated physical and laboratory parameters among adolescent athletes, as well as to investigate their correlation with electrocardiographic (ECG) alterations following a one-year follow-up period.

**Subjects and methods:**

This prospective observational cohort study examined 93 adolescent male athletes (aged 11.6 ± 1.15 years, range 10–14) who underwent baseline health evaluations, including physical examination, laboratory tests [25(OH) vitamin D, parathyroid hormone, and muscle enzyme assays], and cardiovascular (CV) assessment with electrocardiography (ECG). Only patients with normal CV findings were included. Follow-ups were performed after one year.

**Results:**

At follow-up, weight, height, and muscle mass increased significantly, whereas fat mass and systolic pressure decreased. Vitamin D levels declined (21.9 ± 10.8 vs. 29.8 ± 8.6 ng/mL; *p* < 0.001), with compensatory rise in parathyroid hormone (38.8 ± 2.7 vs. 33.9 ± 1.6 pg/mL; *p* < 0.001). Among vitamin D-deficient athletes (*n* = 79), ECG changes occurred in 26.6%, mainly sinus arrhythmia (15/79, 19%) and T-wave inversion (13/79, 16%). Vitamin D deficiency was associated with higher odds of T-wave inversion (OR 4.53; 95% CI 1.11–18.40; *p* = 0.035) and increased prevalence of U-wave and incomplete right bundle branch block.

**Conclusions:**

The drop in vitamin D levels over one year raises concerns, highlighting the need for monitoring and interventions due to vitamin D's role in musculoskeletal and CV health. Regular monitoring and preventive strategies are recommended to maintain optimal vitamin D levels and CV health among young athletes.

## 1 Introduction

Sports are crucial for adolescent development, fostering physical, mental, and social growth through discipline and teamwork. However, adolescent athletes face unique challenges, including rapid physical changes, musculoskeletal risks, and a strong competitive drive. Unhealthy behaviours in sports can have immediate and long-term health consequences, especially in high-level competitions. Thus, maintaining health is key to performance and injury prevention, making health assessments vital for evaluating the well-being of adolescent athletes (1).

Vitamin D is a crucial nutrient for health, fitness, and recovery in sports. While traditionally recognised for its role in maintaining calcium levels, it is now understood to function as a hormone influencing immune defense, protein synthesis, hormone synthesis, and other physiological processes (2).

Vitamin D is an important regulator of musculoskeletal strength, immune defense, and cardiovascular (CV) function. Beyond its traditional role in calcium homeostasis, it functions as a hormone that influences protein synthesis, inflammation, and neuroendocrine regulation (2). It inhibits renin biosynthesis (3) and downregulates proinflammatory T-helper (Th) cytokines, such as Th-1, including interleukins (IL) such as IL-6, IL-8, IL-12, interferon-*γ*, and tumour necrosis factor-α (TNF-α), while promoting anti-inflammatory Th-2 cytokines, such as IL-4, IL-5, and IL-10 (4). Vitamin D deficiency has been associated with metabolic syndrome, myocardial hypertrophy, and both systolic and diastolic dysfunction (5).

Epidemiological studies have confirmed an association between low serum 25-hydroxyvitamin D [25(OH)D] concentrations and CV diseases, including coronary artery disease, hypertension, and arrhythmias (6–8).

Vitamin D is primarily produced through sun exposure, with smaller amounts obtained from dietary sources such as oily fish, egg yolks, and dairy products. Its importance in sports nutrition is increasingly recognised, particularly because of the prevalence of vitamin D deficiency in various athletic activities, which is linked to reduced strength and endurance and a heightened risk of injury (9). The incidence of vitamin D deficiency is notably higher during infancy and adolescence than in later life stages, attributable to the rapid growth and development characteristics of these periods. Consequently, it is crucial to prevent vitamin D deficiency in athletes within these age groups because of its potential adverse effects on health (10). Although the association between vitamin D status and cardiac function has been investigated in adult athletes, there is a paucity of data concerning adolescent populations (5, 11, 12). This study aimed to assess longitudinal changes in vitamin D status among adolescent athletes and determine whether vitamin D deficiency correlates with alterations in electrocardiographic (ECG) parameters that may indicate early electrophysiological changes.

## 2 Subjects and methods

### 2.1 Study design and timing of assessments

This one-year prospective longitudinal study involved adolescent male athletes aged 10–14 years, with 93 participants (mean age 11.6 ± 1.15 years) recruited from local sports clubs in Japan. The inclusion criteria required participants to be in good health, without chronic illnesses or cardiovascular abnormalities. Athletes with health issues at baseline or prior vitamin D supplementation were excluded.

### 2.2 Baseline and follow-up assessments

At the initial baseline visit, all participants underwent a comprehensive health assessment encompassing a detailed physical examination, laboratory testing, and cardiovascular evaluation. One year later, the same individuals participated in a follow-up assessment using the same protocol to assess potential changes in their health parameters over time.

### 2.3 Physical examination (anthropometric/medical measurements)

Physical examination included measurements of weight, height, body mass index (BMI), skeletal muscle mass, fat mass, heart rate, respiratory rate, and blood pressure. Weight, skeletal muscle mass, and fat mass were measured using a “Bioelectrical Impedance Analysis” scale, and height was measured using a stadiometer. Blood pressure was recorded using a standardised automated sphygmomanometer, with systolic (SBP) and diastolic (DBP) values measured after five minutes of rest.

### 2.4 Laboratory investigations

Venous blood samples were obtained after overnight fasting for laboratory analysis. The primary biochemical markers assessed included serum 25-hydroxyvitamin D, parathyroid hormone (PTH), thyroid-stimulating hormone (TSH), and ferritin. These were quantified using a chemiluminescent immunoassay (CLIA). Normal vitamin D, PTH, TSH, and ferritin levels were defined as ≥30 ng/mL, PTH levels as 10–65 0.5–5 mIU/L, and ferritin levels as 30–300 ng/mL.

Baseline assessments of serum calcium, phosphorus, iron, albumin, potassium, and random blood glucose levels were conducted using colorimetric analysis with an automated chemical analyzer. Muscle enzyme levels, specifically serum creatine kinase (CK) and lactate dehydrogenase (LDH) levels, were evaluated using the kinetic method with an automated chemical analyser. A complete blood count (CBC) was performed using a haematology analyser. Vitamin D status was categorized according to the Endocrine Society Clinical Practice Guideline, with deficiency defined as serum 25(OH)D levels below 20 ng/mL, insufficiency as levels between 20 and 29 ng/mL, and sufficiency as levels equal to or exceeding 30 ng/mL (13).

### 2.5 Electrocardiographic evaluation

Standard 12-lead electrocardiograms (ECGs) were acquired at baseline and after one year. ECG parameters, including the PR interval, QRS duration, QT interval, corrected QT (QTc) interval, *P*-wave axis, and T-wave axis were documented. All ECGs were independently evaluated by two blinded, paediatric cardiologists. In cases of disagreement, a senior electrophysiologist made the final decision. ECG findings were interpreted according to the contemporary paediatric athlete ECG interpretation criteria described by Halasz et al. (14), developed in collaboration with the European Society of Cardiology (ESC).

### 2.6 Statistical analysis

The collected data were analysed using descriptive and inferential statistical methods. Data are presented as mean ± standard deviation (SD). The normality of continuous variables and within-subject differences (baseline vs. follow-up) was tested using the Shapiro–Wilk test and visual inspection of histograms and Q–Q plots. Categorical variables were compared using chi-square tests; when the expected cell counts were <5, Fisher's exact test was applied. A paired sample *t*-test was used to compare the baseline and follow-up values for continuous variables. Pearson's correlation was used for the bivariate analysis of the association strength between two variables and the direction of the relationship. Statistical significance was set at *p* < 0.05. All statistical analyses were performed using SPSS (version 26).

Power analysis using G*Power 3.1 (paired-sample *t*-test, *α* = 0.05, power = 0.8) indicated that a minimum of 84 participants was required to detect a medium effect size (*d* = 0.3). The final sample of 93 athletes provided sufficient statistical power for longitudinal comparisons.

To identify variables associated with a high risk of vitamin D deficiency, binary logistic regression was employed to calculate odds ratios (ORs) with a 95% confidence interval and *p*-values, thereby assessing the association between the dependent and independent variables. A receiver operating characteristic (ROC) curve was used to evaluate the diagnostic efficacy of these high-risk variables. The performance of the cutoff value was assessed by calculating Youden's J statistic, with values near “1” indicating a strong performance (J = Sensitivity + Specificity–1).

To assess the impact of vitamin D deficiency on key laboratory parameters, a simple linear regression analysis was performed to determine the regression coefficient, accompanied by a 95% confidence interval and *p*-values. Additionally, to examine the association between vitamin D deficiency and positive electrocardiogram (ECG) changes in athletes following a one-year follow-up, logistic regression analysis was employed to calculate the odds ratio (OR) and 95% confidence interval.

## 3 Results

A cohort of 93 male adolescent athletes aged 10–14 years, with a mean age of 11.6 ± 1.15 years, underwent health assessments at baseline and after a one-year follow-up period. The study identified significant changes in various anthropometric, biochemical, and cardiovascular (CV) parameters.

### 3.1 Anthropometric/medical measurements

The athletes demonstrated significant increases in both weight and height during the one-year follow-up period. Specifically, weight increased from 40.2 ± 6.8 kg to 42.7 ± 9.2 kg (*p* = 0.036), and height increased from 151.4 ± 9.2 cm to 155.4 ± 12.1 cm (*p* = 0.012). Despite these changes, the Body Mass Index (BMI) remained relatively stable, with no significant change observed from 17.4 ± 1.9 to 17.5 ± 2.1 Kg/m² (*p* = 0.731). Additionally, there were significant increases in skeletal muscle mass from 16.6 ± 1.4 to 17.2 ± 1.6 kg (*p* = 0.008), while fat mass significantly decreased from 9.4 ± 0.7 to 8.8 ± 0.8 kg (*p* < 0.001), as shown in Table 1. A significant reduction in Systolic Blood Pressure (SBP) was observed, decreasing from 113.3 ± 9.7 to 109.3 ± 11.4 mmHg (*p* = 0.011). In contrast, Diastolic Blood Pressure (DBP) (70.5 ± 8.5 to 71.5 ± 9.2 mmHg, *p* = 0.441), pulse rate (70.1 ± 8.7–70.8 ± 10.4 beats/min, *p* = 0.622), and respiratory rate (16 ± 2–15 ± 3 breaths/min, *p* = 0.991) did not exhibit significant differences (Table 1).

**Table 1:** Comparison of baseline and one-year follow-up anthropometric and clinical measurements in adolescent athletes.

Anthropometric/Medical Measurements	At baseline	After 1 year	*p*-Value
Weight (Kg)	40.2 ± 6.8	42.7 ± 9.2	0.036*
Height (cm)	151.4 ± 9.2	155.4 ± 12.1	0.012*
BMI (Kg/m^2^)	17.4 ± 1.9	17.5 ± 2.1	0.731
Skeletal muscle mass (Kg)	16.6 ± 1.4	17.2 ± 1.6	0.008*
Fat mass (Kg)	9.4 ± 0.7	8.8 ± 0.8	<0.001*
HR (beat/min)	70.1 ± 8.7	70.8 ± 10.4	0.622
SBP (mmHg)	113.3 ± 9.7	109.3 ± 11.4	0.011*
DBP (mmHg)	70.5 ± 8.5	71.5 ± 9.2	0.441
Respiratory rate (breath/min)	16 ± 2	15 ± 3	0.991

Values are presented as mean ± SD.

BMI, body mass index; HR, heart rate; SBP, systolic blood pressure; DBP, diastolic blood pressure.

* *p* < 0.05 indicates statistical significance.

### 3.2 Laboratory investigations

A concerning finding was the significant reduction in serum vitamin D levels, which decreased from 29.8 ± 8.6 to 21.9 ± 10.8 ng/mL (*p* < 0.001). This 27% reduction indicates a potential risk of vitamin D deficiency among athletes after one year. Additionally, parathyroid hormone (PTH) levels increased significantly (38.8 ± 2.7 vs. 33.9 ± 1.6 pg/mL; *p* < 0.001). Other laboratory parameters, including serum calcium, phosphorus, iron, ferritin, albumin, potassium, muscle enzymes (CK and LDH), thyroid-stimulating hormone (TSH) levels, and complete blood count (CBC) parameters, remained stable, with no significant changes observed (*p* > 0.05), as shown in Table 2.

**Table 2:** Comparison of baseline and one-year follow-up biochemical and haematological parameters in adolescent athletes.

Laboratory investigations	Baseline (Mean ± SD)	After 1 Year (Mean ± SD)	*p*-Value
25(OH) vitamin D (ng/mL)	29.8 ± 8.6	21.9 ± 10.8	<0.001*
Serum calcium (mg/dL)	9.78 ± 0.68	9.86 ± 0.62	0.178
Serum phosphorus (mg/dL)	3.97 ± 0.86	4.1 ± 0.94	0.316
Serum PTH (pg/mL)	33.9 ± 1.6	38.8 ± 2.7	<0.001*
Serum CK (U/L)	93.7 ± 4.5	94.1 ± 4.2	0.813
Serum LDH (U/L)	174.6 ± 6.8	175.1 ± 7.5	0.654
Serum iron (µg/dL)	87.3 ± 21.5	89.6 ± 25.5	0.507
Serum ferritin (ng/mL)	65.4 ± 16.4	68.1 ± 17.1	0.273
Serum albumin (g/dL)	4.39 ± 0.62	4.52 ± 0.58	0.142
Serum potassium (mEq/L)	3.98 ± 0.66	4.11 ± 0.73	0.204
Random blood glucose (mg/dL)	107.6 ± 12.5	110.2 ± 14.6	0.194
TSH (mIU/L)	2.93 ± 0.89	3.11 ± 0.92	0.177
Total leucocyte count ( × 10^3^/µL)	6.10 ± 1.1	6.14 ± 1.5	0.841
Neutrophil ( ×10^3^/µL)	3.14 ± 0.33	3.2 ± 0.4	0.266
Lymphocytes ( ×10^3^/µL)	2.23 ± 0.11	2.19 ± 0.24	0.146
Monocytes (×10^3^/µL)	0.46 ± 0.18	0.44 ± 0.11	0.362
Eosinophils (×10^3^/µL)	0.26 ± 0.09	0.24 ± 0.08	0.111
Basophils (cell/ µL)	8.8 ± 2	9.2 ± 4	0.389
RBC count (×10^6^/µL)	5.34 ± 0.87	5.21 ± 0.54	0.222
Hb (g/dL)	13.5 ± 1.2	13.6 ± 1.5	0.621
Hct (%)	41.09 ± 4.74	40.83 ± 3.14	0.659
MCV (fL)	77.61 ± 9.55	79.11 ± 7.94	0.246
MCH (pg)	25.9 ± 2.16	26.2 ± 1.92	0.318
MCHC (g/dL)	33.2 ± 1.64	32.9 ± 1.44	0.187
RDW (%)	12.31 ± 3.2	11.95 ± 2.3	0.379
Platelet count (×10³/µL)	274.8 ± 47.6	281.5 ± 58.8	0.394
MPV (fL)	7.83 ± 2.9	7.35 ± 3.3	0.293
PDW (%)	17.1 ± 4.13	16.8 ± 3.89	0.611

Values are presented as mean ± SD.

PTH, parathyroid hormone; CK, creatine kinase; LDH, lactate dehydrogenase; TSH, thyroid-stimulating hormone; RBC, red blood cell co unt; Hb, haemoglobin; Hct, haematocrit; MCV, mean corpuscular volume; MCH, mean corpuscular haemoglobin; MCHC, mean corpuscular haemoglobin concentration; RDW, red cell distribution width; MPV, mean platelet volume; PDW, platelet distribution width.

* *p* < 0.05 indicates statistical significance.

### 3.3 Electrocardiographic assessment

Most ECG parameters, including the PR interval, QRS duration, QT interval, and *P*-wave and QRS axes, did not show significant differences (*p* > 0.05) between the two assessments. However, there was a statistically significant reduction in the *T*-wave axis, from 40.5 ± 7.7° to 38.2 ± 6.4° (*p* = 0.028), as shown in Table 3.

**Table 3:** Comparison of baseline and one-year follow-up electrocardiographic (ECG) parameters in adolescent athletes.

ECG Variables	Baseline	After 1 year	*p*-Value
PR interval (ms)	139 ± 27.1	141 ± 22.2	0.583
QRS duration (ms)	88.7 ± 8.9	90.1 ± 7.5	0.248
QT interval (ms)	389.9 ± 23.5	392.8 ± 29.3	0.458
QTc-interval (ms)	413 ± 16.9	410 ± 22.8	0.309
*P*-wave axis (°)	33.9 ± 8.6	32.7 ± 6.9	0.295
QRS axis (°)	63.1 ± 8.2	62.2 ± 9.7	0.495
T-wave axis (°)	40.5 ± 7.7	38.2 ± 6.4	0.028*
R-Voltage in V5 or V6 (mV)	2.37 ± 0.35	2.45 ± 0.55	0.238
S-voltage in V1 or V2 (mV)	1.16 ± 0.28	1.17 ± 0.31	0.818
Voltage sum (mV)	3.5 ± 0.47	3.6 ± 0.65	0.231

Values are presented as mean ± SD.

Clarifications: PR interval, from the onset of the *P*-wave to the beginning of the QRS complex; QTc, corrected QT interval; QRS, ventricular depolarisation axis; R-voltage, amplitude of the R wave; S-voltage, amplitude of the S wave; mV, millivolt; ms, millisecond; voltage sum, R in V5 or V6 + S in V1 or V2; represents combined depolarisation amplitude.

* *p* < 0.05 indicates statistical significance.

### 3.4 Correlation between vitamin D and various anthropometric and biochemical parameters after 1-year follow-up

Significant positive correlations were observed between vitamin D levels and serum calcium and phosphorus levels. In contrast, significant negative correlations were identified between vitamin D and PTH levels, as shown in Table 4. The results of the linear regression analysis were statistically significant. The increase in vitamin D accounted for a 17.5% and 16.7% increase in total serum calcium and serum phosphorus, respectively and *vice versa*, while increase in vitamin D accounted for 3.6% decrease in PTH and *vice versa* (Table 5)

**Table 4:** Correlation matrix between vitamin D and some anthropometric measurements and laboratory investigations in the study participants after 1-year follow-up.

Variable		BMI (Kg/m^2^)	Skeletal muscle mass (Kg)	Fat mass (Kg)	Serum calcium (mg/dL)	Serum phosphorus (mg/dL)	Serum PTH (pg/mL)	Serum CK (U/L)	Serum LDH (U/L)
Vitamin D (ng/mL)	r	−0.168	−0.009	−0.036	0.429	0.408	−0.215	0.054	0.064
	P	0.107	0.931	0.733	<0.001*	<0.001*	0.039*	0.609	0.539

BMI, body mass index; CK, creatine kinase; LDH, lactate dehydrogenase; PTH, parathyroid hormone; p, probability value; r, Pearson's correlation coefficient.

* *p* < 0.05 indicates statistical significance.

**Table 5:** Simple linear regression analysis showing the effect of serum vitamin D on selected biochemical parameters after a one-year follow-up.

Laboratory investigations	B (95% CI)	*β*	SE	*t*-value	R^2^	Adjusted R^2^	*p*-value
Serum calcium (mg/dL)	0.02 (0.012, 0.030)	0.43	0.01	4.54	0.184	0.175	<0.001*
Serum phosphorus (mg/dL)	0.03 (0.016, 0.044)	0.41	0.01	4.27	0.167	0.158	<0.001*
PTH (pg/mL)	−0.05 (–0.090, –0.002)	–0.22	0.02	–2.10	0.046	0.036	0.039*

B, unstandardised regression coefficient; β, standardised regression coefficient; CI, confidence interval; PTH, parathyroid hormone; R^2^, coefficient of determination; SE, standard error.

* *p* < 0.05 indicates statistical significance.

**Figure 1:**
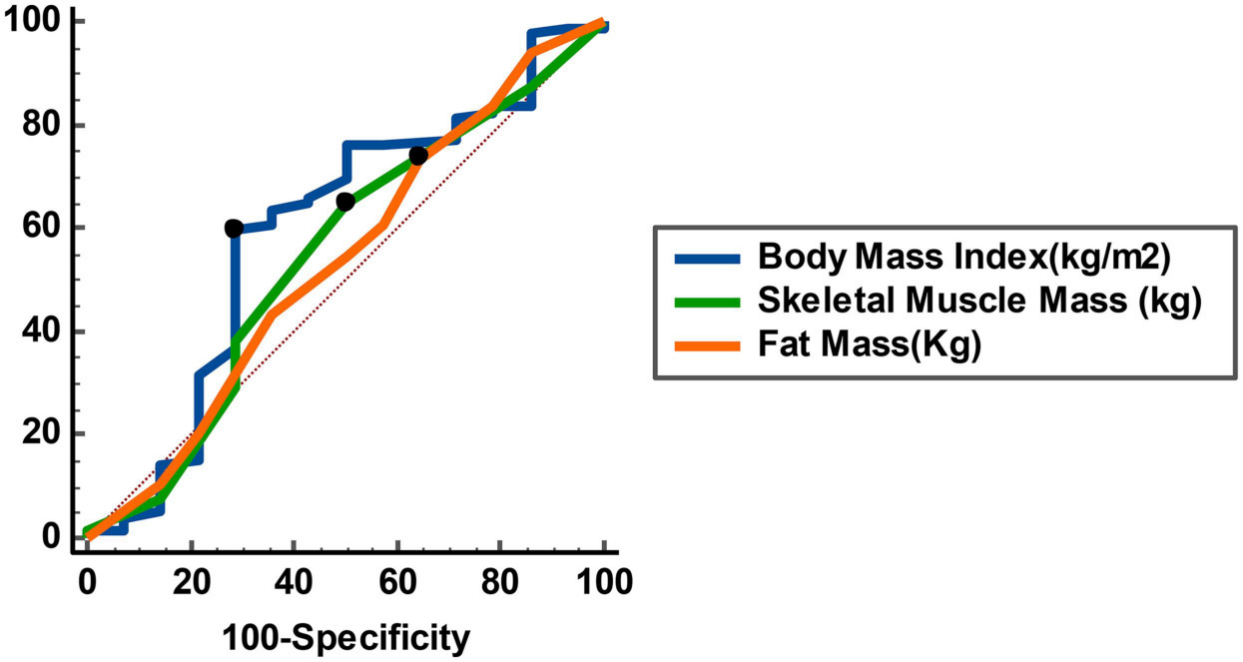
Receiver operating characteristic (ROC) curves of anthropometric variables (body mass index, skeletal muscle mass, and fat mass) for predicting a decline in serum 25-hydroxyvitamin D [25(OH)D] levels after one-year follow-up. The diagonal dashed line represents the line of no discrimination (AUC = 0.5). The corresponding area-under-the-curve (AUC) values were: body mass index = 0.59 (95% CI: 0.42–0.78), skeletal muscle mass = 0.55 (95% CI: 0.37–0.72), and fat mass = 0.54 (95% CI: 0.36–0.70); all non-significant (*p* > 0.05).

The analysis revealed negligible linear negative correlations between body mass index (BMI), skeletal muscle mass, fat mass, and Vitamin D levels. Specifically, a 1 kg/m² increase in BMI corresponded to a 0.016 ng/mL decrease in Vitamin D levels; a 1 kg increase in skeletal muscle mass was associated with a 0.074 ng/mL decrease in Vitamin D; and a 1 kg increase in fat mass resulted in a 0.533 ng/mL decrease in Vitamin D. The multiple linear regression model indicated that approximately 3% of the variance in Vitamin D levels could be attributed to BMI, skeletal muscle mass, and fat mass (R^2^ = 0.030; Table 6). Anthropometric variables with high risk, including BMI, skeletal muscle mass, and fat mass, were identified as having optimal sensitivity (SE) and specificity (SP) for independently predicting a decline in vitamin D levels after a 1-year follow-up, utilising the ROC curve (Table 7, Figure 1).

**Table 6:** Simple and multiple linear regression analyses of anthropometric predictors of serum vitamin D levels after one year of follow-up.

Variable(s)	Simple linear regression				Multiple linear regression		
	B^a^ (95% CI)	β	*t*-value	*p*-value	B^b^ (95% CI)	β	*p*-value
Body-mass index (Kg/m^2^)	−0.016 (–2.257, 0.224)	–0.168	–1.627	0.107	–1.026 (–2.284, 0.231)	–0.170	0.108
Skeletal muscle mass (Kg)	–0.074 (–1.767, 1.619)	–0.009	–0.087	0.931	–0.172 (–1.864, 1.520)	–0.021	0.840
Fat mass (Kg)	–0.533 (–3.621, 2.556)	–0.036	–0.342	0.733	–0.523 (–3.603, 2.557)	–0.035	0.737

B, unstandardised regression coefficient; β, standardised regression coefficient; CI, confidence interval; R², coefficient of determination.

a Crude (simple) regression coefficient.

b Adjusted (multiple) regression coefficient.

No significant associations were observed between vitamin D levels and BMI, skeletal muscle mass, or fat mass (R² = 0.030).

**Table 7:** Receiver operating characteristic (ROC) curve analysis of anthropometric parameters potentially associated with vitamin D decline after a one-year follow-up.

Variable(s)	Cutoff value	Sensitivity (%)	Specificity (%)	Youden index J	AUC (95% CI)	*p*-value
BMI (Kg/m^2^)	≤17.7	59.5	71.4	0.31	0.59 (0.42–0.78)	0.292
Skeletal muscle mass (Kg)	>16	50.0	64.6	0.15	0.55 (0.37–0.72)	0.603
Fat mass (Kg)	≤9.3	73.4	35.7	0.09	0.54 (0.36–0.71)	0.701

AUC, area under the ROC curve; BMI, body mass index; CI, confidence interval; ROC, receiver operating characteristic.

No parameter demonstrated significant discriminatory ability (AUC < 0.6, *p* > 0.05).

### 3.5 Correlation between vitamin D and electrocardiographic variables after 1-year follow-up

There were insignificant positive correlations between vitamin D levels and QRS duration, QT interval, QTc-interval, QRS axis, T-wave axis, and S-voltage in V1 and V2. On the other hand, there were insignificant negative correlations between vitamin D levels and PR interval, *P*-wave axis, R-voltage in V5 or V6, and Voltage sum (Table 8).

**Table 8:** Correlation between serum vitamin D levels and electrocardiographic (ECG) parameters after one-year follow-up.

Variable		PR interval (ms)	QRS duration (ms)	QT interval (ms)	QTc-interval (ms)	*P*-wave axis (°)	QRS axis (°)	T-wave axis (°)	R-Voltage in V5 or V6 (mV)	S-voltage in V1 or V2 (mV)	Voltage sum (mV)
Vitamin D level (ng/mL)	r	−0.189	0.004	0.027	0.049	−0.027	0.139	0.097	−0.119	0.023	−0.080
	P	0.070	0.966	0.794	0.642	0.798	0.183	0.357	0.256	0.828	0.443

No statistically significant correlations were observed between vitamin D levels and ECG intervals, axes, or voltages.

ms, millisecond; mV, millivolt; QTc, corrected QT interval; p, probability value; r, Pearson's correlation coefficient.

Table 9 shows the prevalence of ECG changes associated with vitamin D deficiency after 1-year of follow-up. Using the chi-square test, we found an increased prevalence of ECG changes associated with vitamin D deficiency compared to that in subjects with normal vitamin D levels. There was a significant prevalence of sinus arrhythmia (*p* = 0.002) and T-wave inversion (*p* = 0.012). In contrast, RVCD/incomplete RBBB, U-wave, and T-wave notching were 100% prevalent in patients with vitamin D deficiency compared with those with normal vitamin D levels. This was further confirmed using a logistic regression model (Table 10).

**Table 9:** Comparison of electrocardiographic (ECG) findings between athletes with normal and deficient vitamin D levels after a one-year follow-up.

ECG changes	Groups		*p*-Value
	Normal Vitamin D	Vitamin D Deficiency	
	(*N* = 14)	(*N* = 79)	
Sinus arrhythmia			
No	12 (85.7%)	64 (81.0%)	0.002*
Yes	2 (14.3%)	15 (19.0%)	
RVCD/incomplete RBBB			
No	14 (100%)	77 (97.5%)	0.620
Yes	0 (0.0%)	2 (2.5%)	
U-wave			
No	14 (100%)	66 (83.5%)	0.155
Yes	0 (0.0%)	13 (16.5%)	
Non-specific ST elevation			
No	13 (92.9%)	77 (97.5%)	0.564
Yes	1 (7.1%)	2 (2.5%)	
Non-specific ST depression			
No	13 (92.9%)	78 (98.7%)	0.294
Yes	1 (7.1%)	1 (1.3%)	
T-wave notching			
No	14 (100%)	75 (94.9%)	0.310
Yes	0 (0.0%)	4 (5.1%)	
T-wave inversion			
No	11 (78.6%)	66 (83.5%)	0.012*
Yes	3 (21.4%)	13 (16.5%)	

The *χ*² (chi-square) test was used for comparisons, and Fisher's exact test was applied when the expected cell counts were <5.

N, number of participants; RBBB, right bundle branch block; RVCD, right ventricular conduction delay.

* *p* < 0.05 indicates statistical significance. Values are expressed as numbers (percentages).

**Table 10:** Association between vitamin D deficiency and abnormal electrocardiographic (ECG) findings in athletes after 1-year follow-up.

ECG changes	β	Adjusted OR (95% CI)	*p*-Value	Interpretation
T-wave inversion	1.51	4.53 (1.11–18.40)	0.035*	Significant association with increased risk
RVCD/incomplete RBBB	2.19	8.93 (0.81–98.60)	0.082	Trend toward increased risk
U-wave	2.36	10.60 (0.97–115.30)	0.061	Trend toward increased risk
Sinus arrhythmia	0.29	1.33 (0.25–6.90)	0.675	Non-significant
Non-specific ST elevation	–0.87	0.42 (0.04–4.43)	0.461	Non-significant
Non-specific ST depression	–0.91	0.40 (0.04–4.30)	0.453	Non-significant
T-wave notching	2.02	7.56 (0.65–87.40)	0.111	Non-significant

β, regression coefficient; CI, confidence interval; OR, odds ratio; RBBB, right bundle branch block; RVCD, right ventricular conduction delay.

*p < 0.05 indicates statistical significance. The analysis was performed using Firth's logistic regression.

## 4 Discussion

This longitudinal assessment study examined the health status of adolescent athletes over a one-year follow-up period, focusing on changes identified through comprehensive physical examinations and diagnostic investigations. The findings highlight the significance of regular check-ups in maintaining the health of adolescent athletes and their crucial role in the early detection and prevention of potential health issues. The identification of minor alterations in overall health is particularly important, as these changes can impact both performance and future health of the athletes (15)..

Substantial increases in both weight and height suggest a normal growth trajectory, with the body mass index (BMI) remaining relatively stable despite these changes. The implementation of aerobic and resistance exercises likely contributed to the marked reduction in systolic blood pressure (SBP), indicating enhanced cardiovascular health and a positive training response (16, 17). Diastolic blood pressure (DBP), heart rate, and most laboratory indices showed no significant changes, suggesting stable health conditions (18). While variations in most electrocardiogram (ECG) variables were insignificant, a notable reduction in the T-wave axis was observed in the study. Although the 2° reduction in the T-wave axis reached statistical significance, it remained within the normal adolescent range (30–60°) and likely reflected benign physiological adaptation rather than pathology (19).

Our study showed a positive correlation between vitamin D levels and the QRS axis, as well as negative correlations between vitamin D levels and the PR interval and R voltage. In the same context, intense physical exercise, particularly in running sports, induces cardiovascular modifications that may mimic pathological conditions but actually reflect an organ that has successfully adapted to exercise. Human studies have suggested that vitamin D deficiency negatively affects the structure and function of the heart (11).

In our study, the observed significant reduction of 27% in vitamin D levels among adolescent athletes after one year is concerning, as it contradicts previous studies that reported that regular exercise increases vitamin D levels (20–22). This finding aligns with existing evidence indicating that vitamin D deficiency is prevalent in athletic populations (23–25). This deficiency, particularly noted in athletes, has been associated with increased health risks, as highlighted by de la Puente Yagüe et al., who described it as a global issue, especially problematic during winter when vitamin D synthesis is naturally reduced (12).

Vitamin D deficiency in athletes likely results from multiple factors, including limited ultraviolet-B exposure due to indoor training or practices during non-peak sunlight hours, inadequate dietary intake, and increased physiological demands that deplete the vitamin D stores. Diet, environmental factors, and lifestyle choices, such as avoiding sunlight, further influence vitamin D levels (26–28). For instance, 74.5% of adolescent “Taekwondo” athletes were found to have insufficient vitamin D levels (25). In another study, 70% of athletes were initially deficient but showed increased vitamin D levels after one year due to regular supplementation (29), an intervention not applied to the participants in our study.

Exercise affects vitamin D levels, as physical activity stimulates fat metabolism and releases vitamin D stored in adipose tissue (21, 22). However, the body's increased demand for vitamin D during high-intensity exercise can lead to greater utilization, as it is stored in skeletal muscles rather than remaining in circulation (30).

Vitamin D insufficiency has significant implications, as it can lead to an increased risk of stress fractures, impaired immune function, and delayed recovery, affecting athletic performance (12, 31). Furthermore, it disrupts the balance between calcium and parathyroid hormone (PTH), potentially affecting cardiovascular health (12). Our study demonstrated this through positive correlations between vitamin D and serum calcium and phosphorus levels, and negative correlations between vitamin D and PTH levels. This finding aligns with previous research, which highlighted that vitamin D enhances calcium and phosphate absorption, supports bone mineralisation, and stimulates osteocalcin production. PTH facilitates bone resorption via receptor activator of nuclear factor kappa-B ligand (RANKL) signalling. In addition to inhibiting PTH, vitamin D establishes a feedback loop that promotes bone development and maintains calcium-phosphate equilibrium (32).

Our findings indicate that ECG changes are correlated with vitamin D deficiency after a one-year follow-up period. Guzelcicek et al. (33) identified a correlation between reduced vitamin D levels and modifications in the index of cardioelectrophysiological balance, suggesting that insufficient vitamin D may predispose individuals to arrhythmic risks by influencing cellular repolarization (33). Additionally, Vanga et al. (34) explored the comprehensive role of vitamin D in cardiovascular health and proposed that vitamin D deficiency may exacerbate endothelial dysfunction, enhance inflammatory pathways, and impact cardiac remodelling, all of which could contribute to arrhythmogenesis and adverse cardiac outcomes (34). Previous studies, particularly those by Anees et al. and Canpolat et al., have predominantly focused on atrial arrhythmias linked to vitamin D insufficiency (35, 36). Canpolat et al. identified an elevation in *P*-wave dispersion among vitamin D-deficient individuals; however, no alterations were observed after vitamin D replacement therapy (35).

Numerous studies have investigated the relationship between vitamin D deficiency and ventricular arrhythmias, and have yielded significant findings. A study conducted by Tuliani et al. (37), which included 5,108 participants from the Third National Health and Nutrition Examination Survey (NHANES-III), identified a correlation between vitamin D insufficiency and notable electrocardiographic (ECG) abnormalities (37). Individuals with 25-OH vitamin D levels below 40 ng/mL exhibited major ECG abnormalities that were independently and significantly associated with an increased risk of long-term all-cause combined cardiovascular (CV) events and ischaemic heart disease (37). Vitamin D modulates calcium handling and cardiomyocyte excitability by regulating L-type Ca^2+^ channels, SERCA2a expression, and connexin-43 gap junction coupling. Vitamin D deficiency results in intracellular Ca^2+^ overload, prolonged repolarization, and heightened arrhythmogenic potential (8, 34). Mechanistically, deficiency may contribute to myocardial remodelling through overactivation of the renin–angiotensin–aldosterone system (RAAS), oxidative stress, and endothelial dysfunction. In athletes, insufficient vitamin D levels have been linked to diminished muscle strength, slower recovery rates, and increased injury rates (9). Given that adolescence is a period of rapid skeletal and cardiovascular development, vitamin D deficiency during this stage may have amplified effects (10).

Zhang et al. (21) conducted a comprehensive cross-sectional analysis utilising data from both the NHANES-III and the Atherosclerosis Risk in Communities (ARIC) cohorts, comprising 7,312 participants, and identified no association between serum 25-hydroxyvitamin D levels and QT interval duration, which is in contrast to our findings. Similarly, Tezcan and Tezcan (2025) reported no significant differences in demographic characteristics, laboratory parameters, or QRS-T angles between individuals with and without vitamin D deficiency.

## 5 Strengths and limitations

The primary strength of this study lies in its prospective design, which facilitated monitoring of health changes in adolescent athletes over time. However, this study has several limitations. The sample consisted exclusively of adolescent male athletes, resulting in underrepresentation of females and adults. Additionally, the follow-up period was insufficient to evaluate the long-term effects of athletic training in adolescents. We did not possess quantitative data regarding the training schedule, sun exposure duration, and vitamin D levels in extravascular stores, which are acknowledged determinants of serum vitamin D (27, 28). Future research protocols should incorporate these covariates to enable multivariate adjustment. Confounding factors such as lifestyle and genetics were also uncontrolled. The subgroup analyses related to ECG changes were exploratory and constrained by the small cell counts. Thus, due to sparse data in several rhythm subcategories, Firth's penalized logistic regression was applied to mitigate small-sample bias.

## 6 Implications and future directions

These findings emphasise the need for targeted care in adolescent athletes, who may benefit from regular laboratory tests and adjustments due to the role of vitamin D in musculoskeletal and CV health. Declining vitamin D levels raise concerns regarding bone and immune health, highlighting the importance of tailored management and monitoring. Future research should include balanced gender samples and long-term studies and consider training, diet, and environmental factors. Evaluating interventions, such as vitamin D supplementation, with objective measures, such as advanced imaging, could further enhance our understanding of CV and musculoskeletal health in young athletes. Based on the observed 27% annual decline, we recommend annual vitamin D screening for adolescent athletes, particularly during winter or indoor training seasons. Preventive supplementation of 600–1000 IU daily as per the Endocrine Society Clinical Practice Guidelines (13) and biannual CV evaluation may optimise the musculoskeletal and cardiac health of these patients.

## 7 Conclusion

This study highlights the evolving health profiles of adolescent athletes and the need for customised health promotion strategies. Regular assessments are crucial, as alterations in weight, blood pressure, and declining vitamin D levels have been documented, indicating that early detection and intervention can address emerging health issues. Our findings revealed that vitamin D deficiency was associated with a higher prevalence of certain ECG changes, particularly T-wave inversion, although it was not proven to cause these changes. Proactive monitoring and supplementation of vitamin D, where necessary, is especially critical given its importance in bone health, immunity, and athletic performance. Future research should refine intervention methods and investigate optimal supplementation to better support the health and performance of young athletes.

## Data Availability

The original contributions presented in the study are included in the article/Supplementary Material, further inquiries can be directed to the corresponding author.
